# Synthesis of Carrier-Free Paclitaxel–Curcumin Nanoparticles: The Role of Curcuminoids

**DOI:** 10.3390/bioengineering9120815

**Published:** 2022-12-18

**Authors:** Sena Karaosmanoglu, Yunsen Zhang, Wenli Zhou, Defang Ouyang, Xianfeng Chen

**Affiliations:** 1 School of Engineering, Institute for Bioengineering, University of Edinburgh, Edinburgh EH9 3JL, UK; 2 State Key Laboratory of Quality Research in Chinese Medicine, Institute of Chinese Medical Sciences (ICMS), University of Macau, Macao 999078, China; 3 Department of Medical Oncology, Changzheng Hospital, Naval Medical University, Shanghai 200070, China

**Keywords:** hydrophobic anticancer drugs, curcumin, paclitaxel, carrier-free nanoparticles, self-assembly, molecular modeling

## Abstract

The systemic administration of paclitaxel (PTX)-based combinatorial therapies is significantly restricted due to the multidrug resistance. Curcumin (CUR) not only inhibits cancer-cell proliferation but also reverses the PTX resistance. However, achieving codelivery of these two drugs is a challenge due to their poor water solubility. Herein, we synthesized carrier-free PTX NPs by a facile nanoprecipitation method with the help of CUR and other curcuminoids present in turmeric extract. The prepared NPs demonstrated spherical morphologies with high conformational stability. Experimental studies showed that the presence of both bisdemethoxycurcumin and demethoxycurcumin is essential for the successful formation of spherical and monodisperse NPs. Computational studies revealed that the presence of the more sterically available curcuminoids BMC and DMC makes the self-assembly procedure more adaptable with a higher number of potential conformations that could give rise to more monodisperse PTX-CUR NPs. Compared with PTX alone, PTX-CUR NPs have shown comparable therapeutic efficiency in vitro and demonstrated a higher cellular internalization, highlighting their potential for in vivo applications. The successful formation of PTX-CUR NPs and the understanding of how multiple drugs behave at the molecular level also provide guidance for developing formulations for the synthesis of high-quality and effective carrier-free nanosystems for biomedical applications.

## 1. Introduction

**Figure 1:**
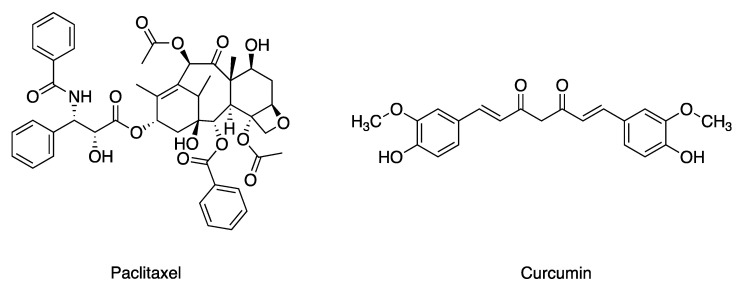
Chemical structures of PTX and CUR. PTX and CUR stand for paclitaxel and curcumin.

Cancer caused the loss of approximately 10 million lives in 2020 alone and it remains the second leading cause of death [1]. The administration of anticancer drugs, chemotherapy, remains as the most common therapy for breast cancer to this day [2]. However, many anticancer drugs are hydrophobic, and this requires the use of organic solvents for their clinical administration, resulting in inefficient therapies and side effects, including cardiotoxicity, nephrotoxicity, neurotoxicity, and hypersensitivity when injected into the bloodstream. To tackle these issues, there has been tremendous research on a variety of carrier-based nanoparticles (NPs), but such strategies often fail to encapsulate drug molecules efficiently and require significant amounts of inorganic and/or organic nanocarriers with potential long-term toxicity. Preparations of carrier-free nanoformulations for the delivery of anticancer drugs with poor water solubility are thus desired [3]. Paclitaxel (PTX, Figure 1), one of the most-prescribed FDA-approved chemotherapeutic agents, prevents microtubule depolymerization and arresting mitosis at the G2/M stages of the cell cycle [3,4]. Despite its effective mechanism, the clinical application of PTX has significant issues due to its low water solubility (0.3 μg/mL) and multidrug resistance (MDR) [5,6,7]. It was reported that the long-term exposure of PTX induced drug resistance via the activation of nuclear factor-κB (NF-κB), resulting in cancer-cell proliferation, invasion, and metastasis [8]. Many studies have demonstrated that the coadministration of curcumin (CUR, Figure 1) with PTX could suppress PTX resistance. CUR, derived from turmeric, *Curcuma longa*, is an anti-inflammatory, antibacterial, and anticancer agent [9]. CUR is known to have synergistic effects when it is combined with anticancer agents [4]. It has been demonstrated that CUR increased the sensitivity of MCF-7 and MDA-MB-231 cells to PTX [10]. In 2020, Saghatelyan et al. reported a phase II clinical study where they investigated the efficacy of CUR in combination with PTX in patients with advanced metastatic breast cancer. The combination treatment was found to be superior compared to PTX and placebo groups in terms of objective response rate and physical performance [11], maintaining its effect even three months after the termination of treatment. Therefore, it would be useful to develop a safe and effective system that could simultaneously deliver PTX and CUR to treat cancer. However, similar to PTX, CUR also has poor water solubility (0.6 µg/mL) and poor bioavailability, which pose a challenge for its clinical application.

To solve the problem, nanotechnology has been studied for the delivery of these two hydrophobic anticancer drugs to reduce their side effects and toxicity and increase the efficiency of the treatments. Such formulations often included carrier-based delivery systems. In 2009, Amiji et al. reported a flaxseed-oil-containing nanoemulsion formulation to encapsulate PTX and CUR to overcome the MDR in ovarian cancer [12]. There have been many carrier-based formulations for the codelivery of PTX and CUR, including polymeric systems [13,14,15], liposomes, niosomes [16], cyclodextrins [17], and graphene oxide [18], but these all suffer from low drug loadings and could pose challenges due to the nontherapeutic carrier mass compositions and the unknown long-term toxicities of the carrier components [3].

To address these issues, mixing two or more hydrophobic drugs together may enable the self-assembly of the drugs into stable NPs without using any carrier components [19,20,21]. Mechanistically, the hydrophobic parts of the molecules interact with each other through noncovalent interactions, such as van der Waals, electrostatic, and induction interactions, which enable the organization of the drug molecules into NPs [3,22,23]. Similar approaches were adopted to facilitate the preparation of carrier-free PTX-containing NPs [24,25,26,27,28]. However, it is highly challenging to produce good-quality carrier-free NPs. The synthesized NPs often have very poor uniformity, irregular shapes, and low stability. Herein, we report an effective formulation for the synthesis of high-quality carrier-free PTX-CUR NPs and then study the mechanism of the successful self-assembly with complimentary experimental and computational studies. The quality of the resulting NPs was explored by changing different variables, including the PTX:CUR weight ratio and the purity of CUR. The prepared NPs were characterized using dynamic light scattering (DLS), transmission electron microscopy (TEM), and ultraviolet visible (UV-Vis) spectroscopy. The computational studies explored the importance of CUR purity in the self-assembly process and provided an understanding of the underlying mechanisms enabling carrier-free NP formation. To the best of our knowledge, this is the first study on the role of curcuminoids in the self-assembly of NPs. The presence of sterically more available bisdemethoxycurcumin (BMC) and demethoxycurcumin (DMC) plays a vital role in the self-assembly process. This study provides a new way to develop CUR-containing nanomedicine formulations, which tackles the water insolubility issue of anticancer drugs and generates synergistic anticancer effects.

## 2. Materials and Methods

### 2.1. Chemicals and Reagents

CUR (purity >98%, >65%), DMC and tetrahydrofuran (THF) were obtained from Sigma Aldrich, UK. PTX and BMC were purchased from Fluorochem (U.K.). 1,2-Distearoyl-sn-glycero-3-phosphoethanolamine-N-[(polyethylene glycol)]-fluorescein (DSPE)-PEG-COOH (5k) was purchased from Creative PEGworks, USA. Dulbecco’s Modified Eagle Medium (DMEM), fetal bovine serum (FBS), trypsin, and penicillin–streptomycin (10,000 U/mL) were obtained from Gibco, UK. Hoechst 33342, 3-(4,5-dimethyl-2-thiazolyl)-2,5-diphenyl-2H-tetrazolium bromide (MTT), and dimethyl sulfoxide (DMSO) were obtained from Sigma Aldrich, UK. All reagents were used without further purification.

### 2.2. Preparation and Characterization of Various PTX-CUR NPs

Carrier-free NP suspensions were prepared using the nanoprecipitation method. First, predetermined solutions of CUR and PTX were prepared in THF, separately, at different concentrations, to achieve the weight ratios stated in Table 1. Next, 100 µL was withdrawn from each of these solutions and mixed to form a 200 µL drug solution with the desired weight ratio, containing 0.2 g of PTX and/or CUR in total. The mixture was then quickly added to 5 mL of deionized water under vigorous stirring to trigger self-assembly. The drug mixture was stirred for 5 min, where 200 µL DPE-PEG-COOH solution in water (0.1 mg/mL) was added one minute before the end of stirring as a surfactant. The NP suspension was sonicated for 20 min. The prepared NPs were stored at 4 °C until further use and characterized by DLS and TEM (JEOL JEM-1400 Plus at Wellcome Trust of Biology Image Centre of Edinburgh). CUR-PTX NPs were purified with ultracentrifuge tubes (MWCO: 5k). The mass spectrometry (MS) analysis of the CUR obtained from turmeric powder was performed using Bruker UK MicroTOF LC MS Neg 50-1000. Standard solutions of PTX and CUR were prepared using stock solutions of 1 mg/mL for each drug molecule in THF. These solutions were then further diluted with water to reach the required concentrations. Nanodrug concentrations were calculated using calibration curves (Supplementary Figure S1) constructed with NanoDrop™ 2000 Spectrophotometer, Thermo Fisher.

### 2.3. Computational Simulations

#### 2.3.1. System Setup of Molecular Dynamics Simulation

PTX-CUR (proportion of molar ratio: 12:27) and PTX-CUR-BMC-DMC (12:19:3:6) system were constructed to mimic the experimental methods of NP formation with 98% and 65% CUR, respectively, and to observe relevant conformation and stability in pure water. All molecular dynamics simulations (MD) were performed using GROMACS 2021.5 [29,30] package under generalized amber forcefield (GAFF) and accelerated high-performance computing center with GPU NVIDIA RTX 3090 and CPU Intel^®^ Core™ i9-10900K @ 3.7 GHz. Restrained ElectroStatic Potential was used as the charges of PTX, CUR, BMC, and DMC from Multiwfn [31] and Gaussian16 with B3YLP/6-311G** level [32,33,34], and relevant topology parameters were generated using Sobtop [35].

#### 2.3.2. Unbiased MD Simulation

An unbiased MD method was employed to describe the self-assembly process of the two NP systems. Initially, molecules were put in a 7.5 × 7.5 × 7.5 nm^3^ box with a water model of tip3p. In the energy minimization step, the conjugate gradient and steep descent algorithm [36] were used to eliminate the irrational contacts of intermolecules and converge the maximum force <100 kJ mol^−1^ nm^−1^. Next, each task was performed in the isothermal−isobaric (NPT) ensemble at 1.0 bar and was coupled isotropically with 4.5 × 10^−5^ compressibility and a coupling constant of 12.0 ps under the Parrinello−Rahman algorithm [37]. The temperature was set at 298.15 K and coupled with a coupling constant of 1.0 ps under v−rescale algorithm [38]. We set 1.0 nm as the cut-off distance for short-range interactions and used the particle-mesh Ewald algorithm [39] to compute the long-range interaction.

NPT simulation (100 ns) for each system in water was executed to generate the trajectory of NP motion and to obtain a stable cluster model. Subsequently, 200 ns simulated annealing [40] of five time- and temperature-points (0 ps, 8 ns, 10 ns, 18 ns, and 20 ns; 0 K, 320 K, 320 K, 0 K, and 0 K) to inspect the structural stability and obtain rational NP conformations in water. Visual molecular dynamics (VMD) [41] and Qtgrace (https://sourceforge.net/projects/qtgrace/, accessed on 1 May 2022) were employed to visualize the MD results.

### 2.4. In Vitro Cytotoxicity Assessment

The cytotoxicity of PTX-CUR NPs was measured by the MTT assay. In general, MCF-7 and MDA-MB-231 cells were cultured in the 96-well plates in medium (200 μL) at 10 × 10^3^ cells/well and 15 × 10^3^ cells/well, respectively, and incubated overnight. Then, the medium was substituted with medium composed of free PTX, CUR, the mixture of PTX and CUR, and PTX-CUR NPs with the final concentrations of 1, 10, 100, and 1000 nM. After incubating for either 48 h or 72 h, cells were incubated with MTT (20 μL, 5 mg/mL) for another 4 h at 37 °C. Hereafter, supernatant was removed, and blue formazan crystal was dissolved by DMSO (150 μL). The absorbance of each well was determined via a microplate reader at 490 nm, and the following formula was used to evaluate the cell inhibition:

$$Cell\;inhibition\;ratio\;\left(\%\right)=\frac{A_{positive}-A_{sample}}{A_{positive}-A_{blank}}\times 100\%$$

The medium without cells or treatments was regarded as a blank group, and cells without drugs were taken as a positive group. For all samples, the cytotoxicity experiment was repeated three times.

### 2.5. In Vitro Cellular Uptake

To investigate the cellular uptake of the released CUR molecules, MCF-7 cells were incubated with the prepared PTX-CUR NPs or free CUR at 37 °C for 4 h. Fluorescence images of the incubated cells were taken using a confocal laser scanning microscope, in which Hoechst 33342 was used to specifically stain the nucleus.

## 3. Results and Discussion

### 3.1. Synthesis of PTX-CUR NPs

The CUR-PTX NPs were prepared using a single-step nanoprecipitation method. The CUR used in these initial studies were obtained from curcuma longa powder (>65% by HPLC). As a starting point for controlling the NP characteristics, we first studied the effect of different weight ratios of PTX and CUR to be able choose the most successful NP formulation. First, standard solutions of CUR and PTX were prepared in THF, separately, at different concentrations to achieve the weight ratios stated in Table 1. Next, 100 µL was withdrawn from each of these solutions and mixed to form a 200 µL drug solution with the desired weight ratio. The mixture was then added dropwisely to 5 mL of deionized water under vigorous stirring to trigger self-assembly. As this study aims to prepare carrier-free NPs, a minimal amount of the PEG was used and the weight ratio between PTX and PEG was kept at 1:0.2, respectively. This is done to make sure that PEG acts as a surfactant, rather than a carrier platform. In addition, if the molecular weight of the DSPE-PEG polymer is considered, 5000 g/mol, relative to PTX’s 853.9 g/mol, the difference in molar ratio comes to 1:0.03 (PTX to PEG). In the formulation, PEG acts as a surfactant to improve the stability of the carrier-free NPs, rather than as a carrier platform. It is worth noting that the PEG molecules were added into the solution after the formation of the pure-drug NPs. Therefore, they are not expected to play any roles in the self-assembly process but at the stabilization of the formed NPs. The NP suspensions were sonicated for a further 20 min and stored at 4 °C for future use.

**Figure 2:**
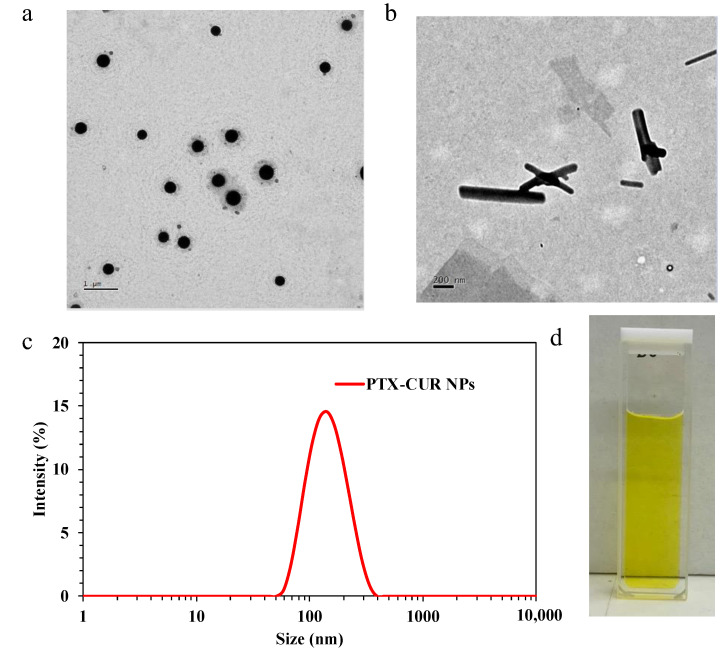
(**a**) TEM image of 1:1 PTX-CUR (*w*/*w*) NPs with 65% CUR—the scale bar is 1 µm; (**b**) TEM image of PTX NPs showing the formation of needle-like aggregates—the scale bar is 200 nm; (**c**) representative DLS size distribution result of 1:1 (*w*/*w*) PTX-CUR NPs with 65%; (**d**) digital image of 1:1 PTX-CUR (*w*/*w*) NP solution in a quartz cuvette. PTX and CUR stand for paclitaxel and curcumin.

The initial DLS results (Table 1) suggested an increase in PTX amount relative to CUR, and vice versa, can rapidly disrupt the self-assembly process. For example, while 1:1, 2:1, and 1:2 weight ratios of CUR:PTX formed relatively narrowly dispersed NPs, increasing either the CUR or PTX weight ratios (1:3 or 3:1) resulted in the formation of visible aggregates on Day 2. Of the successful formulations, the NPs with a 1:1 weight ratio of PTX and CUR exhibited an average size of 162 nm with 0.18 PDI (Figure 2c) and were further characterized using TEM (Figure 2a), where largely symmetric and spherical NPs were imaged. The zeta potential value of 1:1 (*w*/*w*) PTX-CUR NPs was −35.4 mV. When compared to the similar examples from the field of carrier-free nanomedicines, the spherical symmetry of the PTX-CUR NPs is among the best obtained NP morphologies. As a control study, an attempt was also made to prepare PTX NPs with DSPE-PEG-COOH as the surface stabilizer, but without the addition of CUR, using the same method as described above. An average size of 679 nm was observed using DLS, and the subsequent TEM characterization (Figure 2b) revealed the formation of needle-shaped aggregates of the similar size range inside the solution, showing the inability of PTX to self-assemble into NPs in the absence of carrier molecules/platforms. In the literature, dimer molecules of covalently linked PTX were synthesized via dicarboxylic acid linkers to overcome this problem, which contained either an aliphatic carbon chain or a disulfide bond (R: C4, C6, C8, C9, S-S). The prepared dimers (PTX_2_) were then used to form PTX_2_ NPs in an aqueous solution without using any carriers [42].

**Figure 3:**
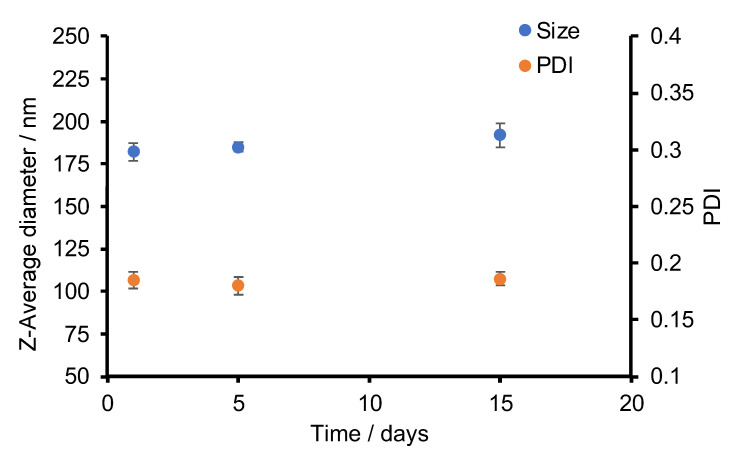
The evolution of size and PDI of 1:1 PTX-CUR NPs over a period of 15 days. PTX and CUR stand for paclitaxel and curcumin.

The stability of the PTX-CUR NPs with a 1:1 weight ratio of PTX and CUR was also monitored using DLS (Figure 3), in which a fresh batch of NPs were prepared and tested for their average sizes and PDIs over a period of 15 days. The samples were stored at 4 °C. While there was a small gradual increase in the NP size, the rise was from ~185 nm to ~195 nm, which can be regarded as a small change. In addition, the PDI values remained low (<0.2) throughout the whole monitoring period.

### 3.2. Curcuminoids and the Self-Assembly Process

With the success of high-quality PTX-CUR NP synthesis with 65% purity CUR, we next decided to test how high-purity CUR (>98%) would behave when combined with PTX. A series of experiments were performed to test the NP synthesis using PTX and high-purity CUR (Table 2). While the nanoprecipitation procedure was kept the same as above, i.e., 1 mg/mL standard THF solutions of both drugs into 5 mL water under vigorous stirring, the results were sharply different. It was discovered that PTX and 98% pure CUR mixtures did not self-assemble into well-controlled NPs in any of the samples. The synthesis led to polydisperse suspensions with visible aggregates after Day 1, even when PEG weight ratios were increased to improve the stability of any potentially formed NPs. The sizes of the formed NPs ranged from 1520 nm to 6150 nm and the PDI values were between 0.65 and 1.00, indicating very polydisperse microsized structures.

**Figure 4:**
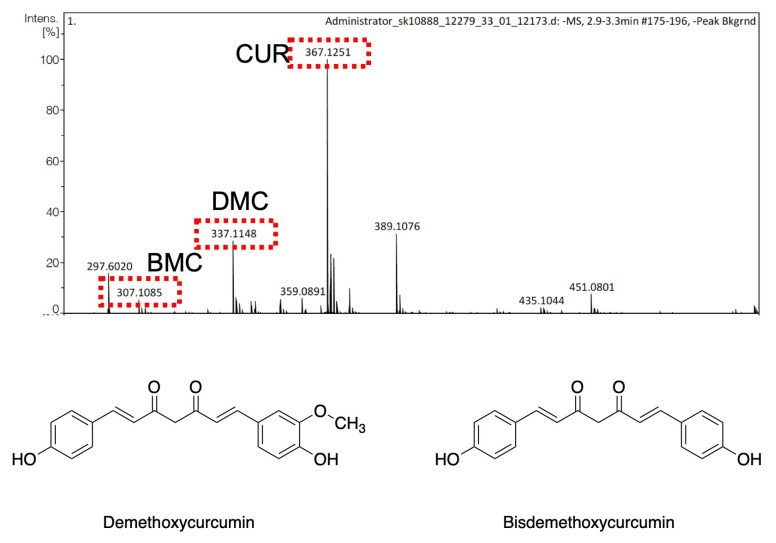
The LC−MS spectra of the >65% CUR product (**top**) and the chemical structures of the identified impurities (**bottom**). CUR stands for curcumin.

The results suggest that the impurities in the >65% CUR might have played roles in the successful self-assembly of the PTX-CUR NPs. Therefore, the low-purity (>65%) CUR product was characterized using MS to identify the components. The performed liquid-chromatography mass spectroscopy (LC-MS) analysis (Figure 4) enabled the identification of the two main impurities as DMC (*m*/*z* 337) and BMC (*m*/*z* 307), alongside CUR (*m*/*z* 367). These identifications are also in line with the literature, where both DMC and BMC contents were previously reported in some commercially available CUR [43]. DMC has one methoxy group on the aryl group (Figure 4), whereas BMC lacks both methoxy groups, which may make it less sterically hindered and more available for intermolecular interactions, such as hydrogen bonding, with PTX and other curcuminoids. BMC is also known to have improved stability in the physiological environment, cellular uptake, and antitumor efficacy compared to CUR [43].

In light of the MS analysis results, we synthesized NPs by mixing these two chemicals with CUR to understand their role on NP formation with PTX (Table 3), using the same protocol used in the preparation of the above-described PTX-CUR NPs. As seen in Table 3, Sample 1 was the best-performing formulation, where the average sizes and the PDI values of the NPs were in agreement with the previous results. The UV-Vis measurements showed the specific absorption peaks of PTX and CUR at 227 nm and 425 nm, respectively (Supplementary Figure S2). In comparison, Samples 2–4 failed to form NPs and visible aggregates were observed from these batches. These results hint that none of the curcuminoids were capable of forming NPs on their own when mixed with PTX using our nanoprecipitation procedure. To facilitate the NP formation, we subsequently tested the formulations containing mixtures of curcuminoids. The results show that using two or more of the CUR analogues could help the self-assembly process, thereby generating NPs with much smaller sizes and significantly lower PDI values. Sample 9, which was formulated to have a similar composition to the 65% CUR obtained from turmeric powder, performed similarly to Sample 1, hinting that this composition is beneficial for the self-assembly of the drug molecules.

### 3.3. Modeling Studies—Molecular Dynamic Simulation to Understand the Mechanism of Self-Assembly of PTX-CUR NPs

#### 3.3.1. Self-Assembly of NPs

Understanding the self-assembly process of small molecules into NPs is often achieved via computational studies. Herein, MD was employed to illustrate the NP formation process, to recognize the mechanistic details of the experimental observations, and to reveal the self-assembly process of the nanosystem. MD is based on Newton’s equations of classical mechanics to simulate the motion processes of molecules, where an integral equation, including the description of bonded and nonbonded interactions, can be swiftly computed using graphics processing units (GPUs) and the location, speed, and forces of particles in the whole system can be calculated and simulated.

**Figure 5:**
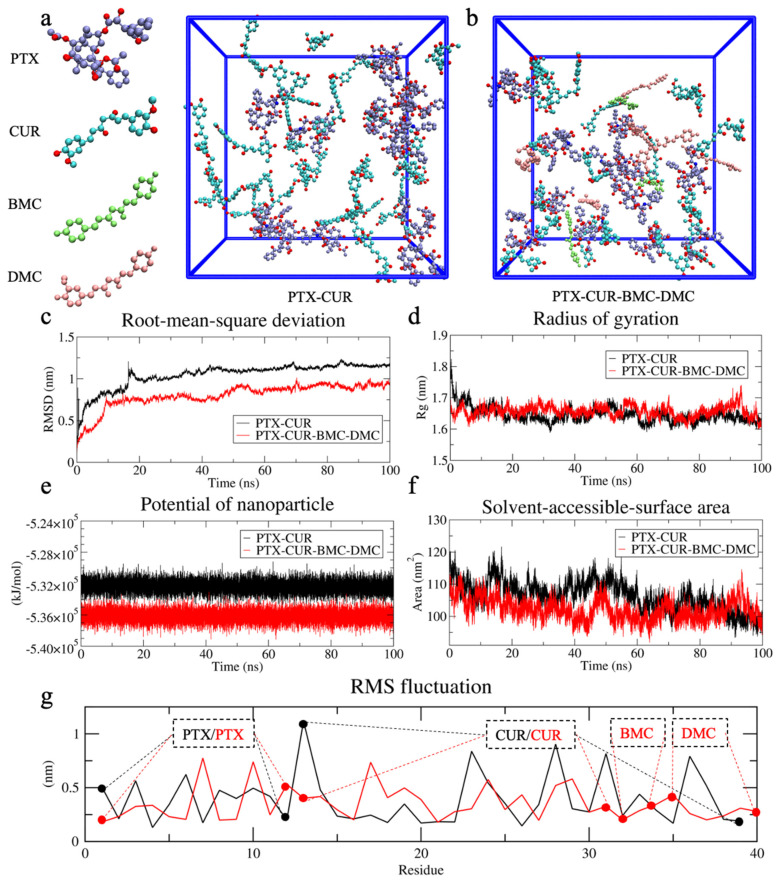
The property representation of NPs, including the initial system state ((**a**) for PTX-CUR and (**b**) for PTX-CUR-BMC-DMC), the profile of RMSD (**c**), Rg (**d**), potential energy (**e**), SASA (**f**), and RMS fluctuation (**g**). PTX, CUR, BMC, and DMC stand for paclitaxel, curcumin, bisdemethoxycurcumin, and demethoxycurcumin, respectively (The black and red solid lines stand for the molecular fluctuation in only PTX-CUR and PTX-CUR-BMC-DMC NPs, respectively. The dotted line defines the number and range of each molecule).

Initially, the starting systems were constructed by placing molecules randomly into box systems, as shown in Figure 5. The root–mean–square deviation (RMSD) was then used to measure the position deviations of specific particles during a 100 ns simulation. RMSD is an indicator for the equilibrium state, and smaller RMSD values were observed for the PTX-CUR-BMC-DMC system (Figure 5c) than for the PTX-CUR, indicating the more stable nature of the formed NPs. In both systems, the radius of gyration (R_g_), which is used to probe size changes during the motion process, was calculated as ~1.65 nm (Figure 5d). In addition, the solvent-accessible surface area (SASA) was used to evaluate the hydrophobic interaction of the NPs. The smaller SASA again pointed towards the more stable nature of the PTX-CUR-BMC-DMC NPs (Figure 5e,f). We further explored the fluctuations of each molecule in systems (PTX-CUR: 1–12, 13–39; PTX-CUR-BMC-DMC: 1–12, 13–31, 32–34, 35–40) to describe the stability distribution in the presence of BMC (32–34) and DMC (35–40) (Figure 5g). Apparently, the replacement of CUR with BMC and DMC reduced the fluctuation of the system and made the remaining CUR more stable.

#### 3.3.2. Representative Conformation Analysis

**Figure 6:**
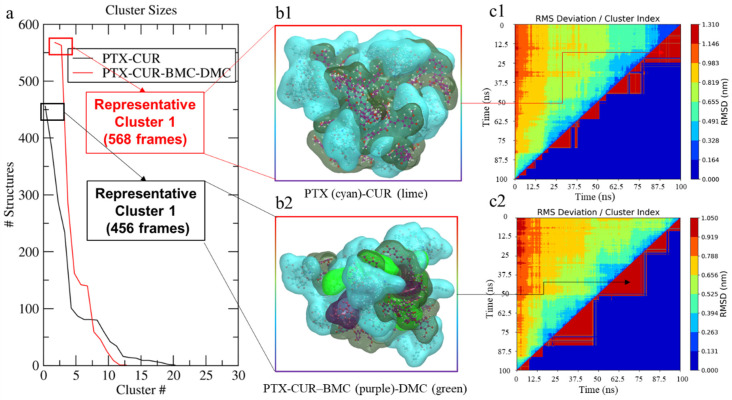
Representative conformation analysis of PTX-CUR and PTX-CUR-BMC-DMC NPs. (**a**) indicates the number of NP conformations (y axis) and clusters (x axis) from the total conformations during the whole simulation process; (**b**) describes the representative structure of NPs in water solvent ((**b1**,**b2**) indicated the PTX-CUR and PTX-CUR-BMC-DMC NPs, respectively); (**c**) denotes the clustering matrix of NPs. PTX, CUR, BMC, and DMC stand for paclitaxel, curcumin, bisdemethoxycurcumin, and demethoxycurcumin, respectively ((**c1**,**c2**) indicated the PTX-CUR and PTX-CUR-BMC-DMC NPs, respectively).

**Figure 7:**
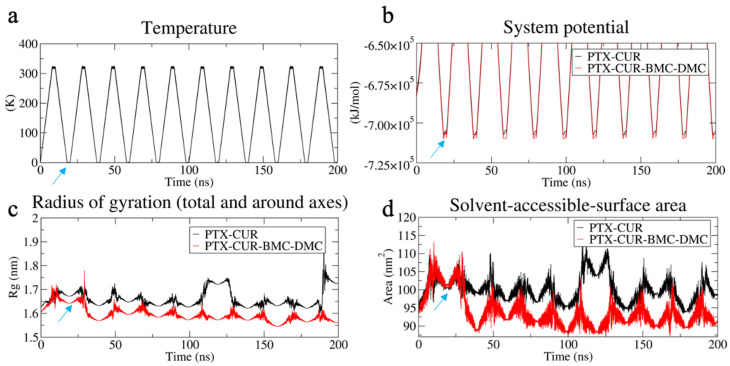
Simulated annealing method revealed the NP size and stability analysis of PTX-CUR and PTX-CUR-BMC-DMC. (**a**) indicates the change in the system temperature from 0 to 320 K under 10-turn simulated annealing. (**b**–**d**) indicate the potential-energy fluctuation, Rg, and SASA during the simulated annealing process, respectively. PTX, CUR, BMC, and DMC stand for paclitaxel, curcumin, bisdemethoxycurcumin, and demethoxycurcumin, respectively. The arrows in figures indicate the first time-point of whole system to reach the 0 K.

Generated NP conformations were then used to analyze the potential representative conformations of the two kinds of NPs in water. When an RMSD cut-off of 0.40 nm was applied, the PTX-CUR-BMC-DMC (corresponding to the NPs from 65% CUR and PTX) system generated a maximum of 568 structures in the representative cluster 1 (total of 12 clusters, red line in Figure 6a) compared to the 458 structures in the representative cluster 1 (total of 21 clusters, black line in Figure 7a) recorded with the PTX-CUR (corresponding to the NPs from 98% CUR and PTX) system (Figure 6). The key point is to understand the success and failure of NPs with different purities. From the perspective of a simulation, the self-assembly (microscopic) of molecules is not equal to the generation of NPs (macroscopic), but we can hypothesize that a more stable process of self-assembly of molecules indicates NPs with better properties. Figure 6b1 and 6b2 reveals the representative conformations (b1 and b2 indicated the PTX-CUR and PTX-CUR-BMC-DMC NPs, respectively). Figure 6c1 and 6c2 reveals clusters distribution in the self-assembly process (c1 and c2 indicated the PTX-CUR and PTX-CUR-BMC-DMC NPs, respectively). The results suggest that the PTX-CUR-BMC-DMC system is likely to generate more collective structures in the modeling studies due to there being more structures in the representative cluster and a smaller number of total clusters. Representative conformations can also be used to assess the formability of the NP systems and given the higher number of possible structures in clusters, the NP system with PTX-CUR-BMC-DMC is more formable compared to the PTX-CUR system. The higher number of conformations may stem directly from the decreased steric hinderance on the curcuminoids BMC and DMC, which can enable different confirmations to form and sustain NPs. Therefore, from the process of micro-self-assembly, the PTX-CUR NP is not as stable as the PTX-CUR-BMC-DMC NP. The rational extension to the process of macro-nanoparticle generation indicates why the synthesis of PTX-CUR (>98%) NPs failed.

#### 3.3.3. Evaluation of System Stability

The stability of the generated NPs was tested in unstable environments by introducing the simulated annealing method. The temperature of the system was varied between 0 K and 320 K in every 10 ns. The changes in potential energy, R_g_, and SASA were observed for both drug-combination systems with the temperature fluctuations (Figure 7). Both R_g_ and SASA decreased at the minimum temperature point (blue arrows in the Figure 7), and this reflects more stable NP structures at lower temperatures owing to a lower system potential. When comparing the NP systems, PTX-CUR-BMC-DMC mostly resulted in lower R_g_ sizes and a smaller SASA in the annealing simulations, further confirming the more stable nature of these NPs.

### 3.4. In Vitro Cytotoxicity

**Figure 8:**
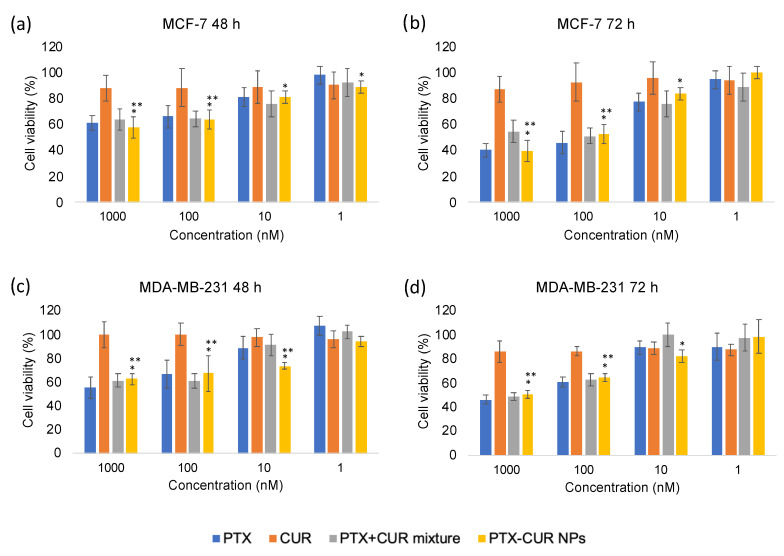
In vitro anticancer activity of PTX, CUR, PTX + CUR mixture, and PTX-CUR NPs in MCF-7 and MDA-MB-231 cells. PTX and CUR stand for paclitaxel and curcumin. Data are presented as means ± SD. * *p* < 0.05 vs. control. ** *p* < 0.05 vs. CUR.

The cytotoxicity of PTX-CUR NPs was measured via the MTT assay on MCF-7 and MDA-MB-231 cells. As depicted in Figure 8, the cytotoxicity of PTX, PTX + CUR, and PTX-CUR NPs increased in a drug-concentration- and incubation-time-dependent manner. The PTX + CUR mixture showed a cell viability of 60% in MCF-7 cells at 48 h and PTX-CUR NPs exhibited a relatively similar cell viability for the same PTX concentration (1000 nM for PTX). The cell viabilities at 72 h decreased to approximately ~45% for the PTX + CUR mixture and the PTX-CUR NP-treated groups. Similar results were also observed in MDA-MB-231 cells. The similar cytotoxicities of the NPs and the drug mixture are attributed to the ability of the free drug molecules to quickly enter cells by diffusion in in vitro studies [19,28,44]. However, therapeutic efficacy is expected to be much better than the mixture in vivo due to the advantages of nanomedicine. For example, in one particular study, PTX-loaded NPs showed superior anticancer efficacy in vivo compared to free PTX alone, but demonstrated weaker efficacy in vitro [44]. Moreover, the cells incubated with free CUR alone showed little-to-no decrease in cell viability due to its low concentration. Overall, it was demonstrated that PTX-CUR NPs showed comparable cytotoxicity to the free PTX + CUR mixture, highlighting their strong potential for the codelivery of PTX and CUR as carrier-free nanodrugs.

To be able to investigate the potential of PTX-CUR NPs for clinic use, we also attempted to increase their concentration. The final nanodrug solution had an estimated concentration of 20 µg/mL PTX and 20 µg/mL CUR as they were prepared using 1 mg/mL drug-stock solutions with 5 mL water. To increase the concentration and make the NPs more suitable for clinical applications, a dialysis-based procedure was performed using Amicon^®^ ultra centrifugal filter tubes (cut-off 10 kDa). The prepared PTX-CUR NP suspensions were added into the Amicon tubes, and they were spun at 3000 rpm for 20 min. After the centrifugation, the concentration of the nanodrug solution increased by 2.6-fold (52 µg/mL), as characterized by the UV-Vis spectrophotometer quantification method. The concentrated NPs showed good stability. Furthermore, as an alternative to the usual maximum-tolerated-dosage chemotherapy plan, a metronomic method with a lower and more frequent dosing schedule has recently emerged. In a 2022 article, it was demonstrated that low and more-frequent doses of Doxil (doxorubicin-loaded liposomes), compared to high and less-frequent doses, exhibited enhanced antitumor effects either alone or in combination with immunotherapy, decreased tumor stiffness, and improved perfusion [45].

### 3.5. Cellular Uptake

**Figure 9:**
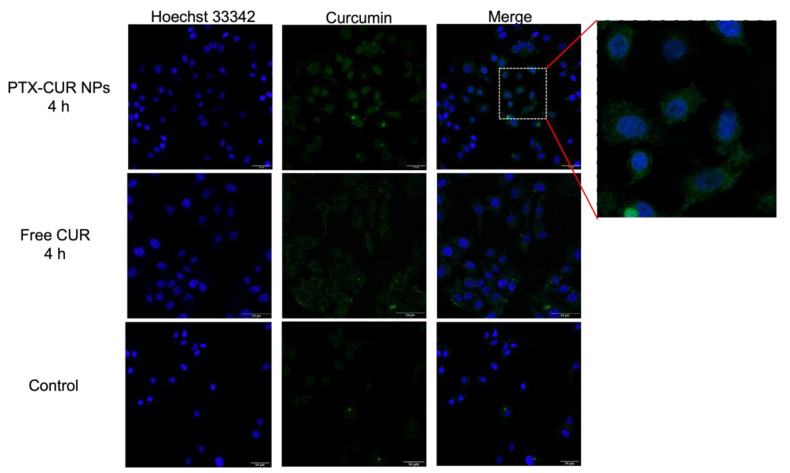
Confocal laser scanning microscopy images of MCF-7 cells after incubation with PTX-CUR NPs for 4 h. Scale bars are 54 μm.

Sufficient cell internalization is desired to achieve an effective therapeutic agent. Therefore, the cellular-uptake behavior of PTX-CUR NPs was evaluated by confocal microscopy. After the incubation of MCF-7 cells with PTX-CUR NPs for 4 h, the nuclei were labeled with Hoechst dye. Compared with the cancer cells treated with free CUR, the PTX-CUR NP-treated group exhibited a stronger fluorescence intensity (Figure 9). This indicates a higher internalization efficiency of PTX-CUR NPs by cancer cells, further demonstrating the potential of the prepared NPs for future in vivo application.

## 4. Conclusions

A nanoprecipitation method was developed to obtain pure-drug PTX-CUR NPs, formed solely by intermolecular physical interactions. Upon studying the self-assembly process, the role of curcuminoids in the CUR mixture was discovered. Experimental studies showed that the presence of both BMC and DMC is essential for the successful formation of spherical and monodisperse NPs. Computational studies further revealed that the presence of the more sterically available curcuminoids BMC and DMC makes the self-assembly procedure more adaptable, with a higher number of potential conformations that could give rise to NPs. PTX-CUR NPs showed comparable therapeutic efficacy in vitro compared to free drugs. Further studies are needed to determine the stability and release profiles of the drugs in biologically relevant media. Curcuminoids are well-known for their accessibility, low cost, safety, and anticancer properties. Various scientific and clinical studies, however, have revealed that curcumin has a limited efficacy because of its low solubility, rapid rate of metabolism, poor bioavailability, and pharmacokinetics. Both BMC and DMC are naturally present in turmeric extract and can be used to improve the quality of many existing curcumin-containing nanoformulations. Our developed method may aid in tunable drug formulations to form nanomedicines with different anticancer drugs, solving their water insolubility issue and contributing to synergistic anticancer effects.

## Figures and Tables

**Table 1:** The effects of different weight ratios of PTX and CUR on the preparation of NPs. PTX and CUR stand for paclitaxel and curcumin.

Weight Ratio		Average Size (nm)	PDI	Appearance at Day 2
CUR	PTX			
1	0	189 ± 2.56	0.13 ± 0.003	Clear
0	1	679 ± 87	0.52 ± 0.18	Large aggregation
1	1	162 ± 11.2	0.18 ± 0.002	Clear
1	2	119 ± 18	0.21 ± 0.003	Clear
1	3	300 ± 34.7	0.45 ± 0.04	Large aggregation
2	1	124 ± 28.2	0.15 ± 0.1	Clear
3	1	222 ± 48	0.30 ± 0.14	Some aggregation

**Table 2:** The size and PDI of PTX-CUR NPs synthesized from PTX and high-purity CUR (>98%). PTX and CUR stand for paclitaxel and curcumin.

CUR Purity	W_PTX_/W_PEG_	Average Size (nm)	PDI
>98%	1:0.1	1520 ± 264	1.00
	1:0.5	2160 ± 396	1.00
	1:1	6150 ± 323	0.70 ± 0.15
	1:2	2490 ± 100	0.65 ± 0.06
	1:5	2900 ± 283	1.00

**Table 3:** The hydrodynamic diameter of the NPs obtained from different drug formulations measured by DLS. The weight ratios are stated in relative to PTX. PTX, CUR, BMC, and DMC stand for paclitaxel, curcumin, bisdemethoxycurcumin, and demethoxycurcumin, respectively.

Sample	NP Composition (Weight Ratios)	Average Size/nm	PDI
1	PTX and 65% pure CUR (1:1)	126 ± 4.25	0.19 ± 0.002
2	PTX and 98% pure CUR (1:1)	4020 ± 84	1.00
3	PTX and BMC (1:1)	3870 ± 67	1.00
4	PTX and DMC (1:1)	5920 ± 195	1.00
5	PTX, BMC, and DMC (1:0.5:0.5)	208 ± 22.6	0.52 ± 0.1
6	PTX, 98% CUR, and BMC (1:0.5:0.5)	290 ± 36.84	0.48 ± 0.03
7	PTX, 98% CUR, and DMC (1:0.5:0.5)	207 ± 27.02	0.40 ± 0.02
8	PTX, 98% CUR, BMC, and DMC (1:0.33:0.33:0.33)	223 ± 33.20	0.42 ± 0.1
9	PTX, 98% CUR, BMC, and DMC(1:0.7:0.1:0.2)	160 ± 3.06	0.20 ± 0.007

## Data Availability

Not applicable.

## Supplementary Materials

The following supporting information can be downloaded at: https://www.mdpi.com/article/10.3390/bioengineering9120815/s1, Figure S1: UV-Vis spectra of PTX and CUR molecules in water at different concentrations and the resulting standard curves; Figure S2. The UV-Vis absorption spectra of free PTX and the synthesized NPs from mixtures of PTX and 65% CUR (1), PTX, BMC, and DMC (5), PTX, 98% CUR, and BMC (6). PTX, CUR, BMC, and DMC stand for paclitaxel, curcumin, bisdemethoxycurcumin, and demethoxycurcumin, respectively.

## References

[1] Xue P., Wang J., Han X.F., Wang Y.J. (2019). Hydrophobic drug self-delivery systems as a versatile nanoplatform for cancer therapy: A review. Colloids Surf. B-Biointerfaces.

[2] Yu F.Z., Tu Y.L., Luo S.W., Xiao X., Yao W., Jiang M.L., Jiang X.Q., Yang R.M., Yuan Y.Y. (2021). Dual-drug backboned polyprodrug with a predefined drug combination for synergistic chemotherapy. Nano Lett..

[3] Karaosmanoglu S., Zhou M., Shi B., Zhang X., Williams G.R., Chen X. (2021). Carrier-free nanodrugs for safe and effective cancer treatment. J. Control. Release.

[4] Abouzeid A.H., Patel N.R., Torchilin V.P. (2014). Polyethylene glycol-phosphatidylethanolamine (PEG-PE)/vitamin E micelles for co-delivery of paclitaxel and curcumin to overcome multi-drug resistance in ovarian cancer. Int. J. Pharm..

[5] Lee S.C., Huh K.M., Lee J., Cho Y.W., Galinsky R.E., Park K. (2007). Hydrotropic polymeric micelles for enhanced paclitaxel solubility: In vitro and in vivo characterization. Biomacromolecules.

[6] Yao Q., Gutierrez D.C., Hoang N.H., Kim D., Wang R.N., Hobbs C., Zhu L. (2017). Efficient codelivery of paclitaxel and curcumin by novel bottlebrush copolymer-based micelles. Mol. Pharm..

[7] Skwarczynski M., Hayashi Y., Kiso Y. (2006). Paclitaxel prodrugs: Toward smarter delivery of anticancer agents. J. Med. Chem..

[8] Wei Y.M., Pu X.L., Zhao L. (2017). Preclinical studies for the combination of paclitaxel and curcumin in cancer therapy. Oncol. Rep..

[9] Aggarwal B.B., Shishodia S., Takada Y., Banerjee S., Newman R.A., Bueso-Ramos C.E., Price J.E. (2005). Curcumin suppresses the paclitaxel-induced nuclear factor-kappa B pathway in breast cancer cells and inhibits lung metastasis of human breast cancer in nude mice. Clin. Cancer Res..

[10] Quispe-Soto E.T., Calaf G.M. (2016). Effect of curcumin and paclitaxel on breast carcinogenesis. Int. J. Oncol..

[11] Saghatelyan T., Tananyan A., Janoyan N., Tadevosyan A., Petrosyan H., Hovhannisyan A., Hayrapetyan L., Arustamyan M., Arnhold J., Rotmann A.-R. (2020). Efficacy and safety of curcumin in combination with paclitaxel in patients with advanced, metastatic breast cancer: A comparative, randomized, double-blind, placebo-controlled clinical trial. Phytomedicine.

[12] Ganta S., Amiji M. (2009). Coadministration of paclitaxel and curcumin in nanoemulsion formulations to overcome multidrug resistance in tumor cells. Mol. Pharm..

[13] Xiong K., Zhang Y., Wen Q., Luo J., Lu Y., Wu Z., Wang B., Chen Y., Zhao L., Fu S. (2020). Co-delivery of paclitaxel and curcumin by biodegradable polymeric nanoparticles for breast cancer chemotherapy. Int. J. Pharm..

[14] Thulasidasan A.K.T., Retnakumari A.P., Shankar M., Vijayakurup V., Anwar S., Thankachan S., Pillai K.S., Pillai J.J., Nandan C.D., Alex V.V. (2017). Folic acid conjugation improves the bioavailability and chemosensitizing efficacy of curcumin-encapsulated PLGA-PEG nanoparticles towards paclitaxel chemotherapy. Oncotarget.

[15] Cui Y., Zhang M., Zeng F., Jin H.Y., Xu Q., Huang Y.Z. (2016). Dual-targeting magnetic plga nanoparticles for codelivery of paclitaxel and curcumin for brain tumor therapy. ACS Appl. Mater. Interfaces.

[16] Alemi A., Reza J.Z., Haghiralsadat F., Jaliani H.Z., Karamallah M.H., Hosseini S.A., Karamallah S.H. (2018). Paclitaxel and curcumin coadministration in novel cationic PEGylated niosomal formulations exhibit enhanced synergistic antitumor efficacy. J. Nanobiotechnol..

[17] Boztas A.O., Karakuzu O., Galante G., Ugur Z., Kocabas F., Altuntas C.Z., Yazaydin A.O. (2013). Synergistic interaction of paclitaxel and curcumin with cyclodextrin polymer complexation in human cancer cells. Mol. Pharm..

[18] Asgari S., Pourjavadi A., Setayeshmehr M., Boisen A., Ajalloueian F. (2021). Encapsulation of drug-loaded graphene oxide-based nanocarrier into electrospun pullulan nanofibers for potential local chemotherapy of breast cancer. Macromol. Chem. Phys..

[19] Yu C.T., Zhou M.J., Zhang X.J., Wei W.J., Chen X.F., Zhang X.H. (2015). Smart doxorubicin nanoparticles with high drug payload for enhanced chemotherapy against drug resistance and cancer diagnosis. Nanoscale.

[20] Zhou M., Zhang X., Yang Y., Liu Z., Tian B., Jie J., Zhang X. (2013). Carrier-free functionalized multidrug nanorods for synergistic cancer therapy. Biomaterials.

[21] Qin S.Y., Peng M.Y., Rong L., Li B., Wang S.B., Cheng S.X., Zhuo R.X., Zhang X.Z. (2015). Self-defensive nano-assemblies from camptothecin-based antitumor drugs. Regen. Biomater..

[22] Grimme S. (2008). Do special noncovalent pi-pi stacking interactions really exist?. Angew. Chem.-Int. Ed..

[23] Emamian S., Lu T., Kruse H., Emamian H. (2019). Exploring Nature and Predicting Strength of Hydrogen Bonds: A Correlation Analysis Between Atoms-in-Molecules Descriptors, Binding Energies; Energy Components of Symmetry-Adapted Perturbation Theory. J. Comput. Chem..

[24] Zhang C.Y., Long L., Xiong Y., Wang C.P., Peng C.P., Yuan Y.C., Liu Z., Lin Y., Jia Y., Zhou X. (2019). Facile engineering of indomethacin-induced paclitaxel nanocrystal aggregates as carrier-free nanomedicine with improved synergetic antitumor activity. ACS Appl. Mater. Interfaces.

[25] Feng B., Niu Z.F., Hou B., Zhou L., Li Y.P., Yu H.J. (2020). Enhancing triple negative breast cancer immunotherapy by ICG-templated self-assembly of paclitaxel nanoparticles. Adv. Funct. Mater..

[26] Lin J.F., Li C., Guo Y., Zou J.J., Wu P.Y., Liao Y.Q., Zhang B., Le J., Zhao R., Shao J.-W. (2019). Carrier-free nanodrugs for in vivo NIR bioimaging and chemo-photothermal synergistic therapy. J. Mater. Chem. B.

[27] Guo Y., Jiang K., Shen Z.C., Zheng G.R., Fan L.L., Zhao R.R., Shao J.W. (2017). A small molecule nanodrug by self-assembly of dual anticancer drugs and photosensitizer for synergistic near-infrared cancer theranostics. ACS Appl. Mater. Interfaces.

[28] Zuo S.T., Wang Z.Y., An X.Q., Wang J., Zheng X., Shao D., Zhang Y. (2021). Self-assembly engineering nanodrugs composed of paclitaxel and curcumin for the combined treatment of triple negative breast cancer. Front. Bioeng. Biotechnol..

[29] Abraham L., van der Spoel H. (2020). GROMACS 2020 Source Code, Version 2020.

[30] Van der Spoel D., Lindahl E., Hess B., Groenhof G., Mark A.E., Berendsen H.J.C. (2005). GROMACS: Fast, flexible, and free. J. Comput. Chem..

[31] Lu T., Chen F.W. (2012). Multiwfn: A multifunctional wavefunction analyzer. J. Comput. Chem..

[32] Stephens P.J., Devlin F.J., Chabalowski C.F., Frisch M.J. (1994). Ab-initio calculation of vibrational absorption and circular-dichroism spectra using density-functional force-fields. J. Phys. Chem..

[33] Krishnan R., Binkley J.S., Seeger R., Pople J.A. (1980). Self-consistent molecular-orbital methods. XX. A basis set for correlated wave-functions. J. Chem. Phys..

[34] Frish M.J., Trucks G.W., Schlegel H.B., Scuseria G.E., Robb M.A., Cheeseman J.R., Scalmani G., Barone V., Petersson G.A., Nakatsuji H. (2016). Gaussian 16.

[35] Sobtop, Version 1.0 (dev2); Tian Lu, S. http://sobereva.com/soft/Sobtop.

[36] Payne M.C., Teter M.P., Allan D.C., Arias T.A., Joannopoulos J.D. (1992). Iterative minimization techniques for ab initio total-energy calculations: Molecular dynamics and conjugate gradients. Rev. Mod. Phys..

[37] Parrinello M., Rahman A. (1981). Polymorphic transitions in single-crystals—A new molecular dynamics method. J. Appl. Phys..

[38] Bussi G., Donadio D., Parrinello M. (2007). Canonical sampling through velocity rescaling. J. Chem. Phys..

[39] Darden T., York D., Pedersen L. (1993). Particle mesh ewald—An N.Log(N) method for ewald sums in large systems. J. Chem. Phys..

[40] Kirkpatrick S., Gelatt C.D., Vecchi M.P. (1983). Optimization by simulated annealing. Science.

[41] Humphrey W., Dalke A., Schulten K. (1996). VMD: Visual molecular dynamics. J. Mol. Graph. Model..

[42] Pei Q., Hu X.L., Liu S., Li Y., Xie Z.G., Jing X.B. (2017). Paclitaxel dimers assembling nanomedicines for treatment of cervix carcinoma. J. Control. Release.

[43] Wunsche S., Yuan L.N., Seidel-Morgenstern A., Lorenz H. (2021). A contribution to the solid state forms of bis(demethoxy)curcumin: Co-crystal screening and characterization. Molecules.

[44] Fan L., Wang J.Q., Xia C.W., Zhang Q., Pu Y.M., Chen L., Chen J.F., Wang Y.X. (2020). Glutathione-sensitive and folate-targeted nanoparticles loaded with paclitaxel to enhance oral squamous cell carcinoma therapy. J. Mater. Chem. B.

[45] Mpekris F., Voutouri C., Panagi M., Baish J.W., Jain R.K., Stylianopoulos T. (2022). Normalizing tumor microenvironment with nanomedicine and metronomic therapy to improve immunotherapy. J. Control. Release.

