# Supplementary material for: Synthesis of Carrier-Free Paclitaxel–Curcumin Nanoparticles: The Role of Curcuminoids
Source: Bioengineering (Basel). 2022 Dec 18;9(12):815. doi: 10.3390/bioengineering9120815 (PMC9774928; doi:10.3390/bioengineering9120815)
Supplement: Supplementary file 1 [file bioengineering-09-00815-s001.zip › bioengineering-2082426-supplementary.pdf]

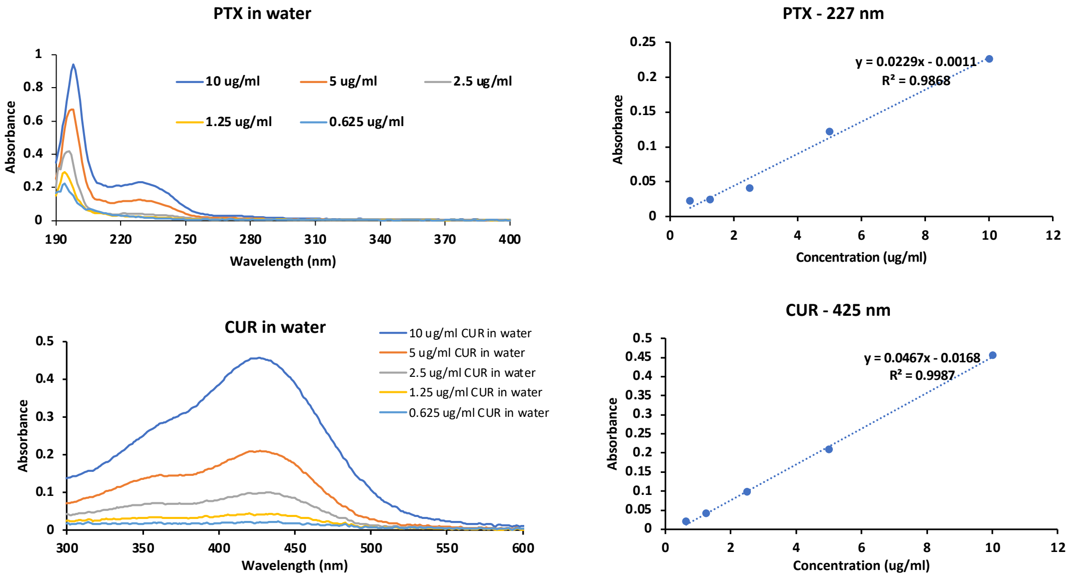

Figure S1. UV-Vis spectra of PTX and CUR molecules in water at different concentrations and the resulting standard curves. PTX and CUR stand for paclitaxel and curcumin.

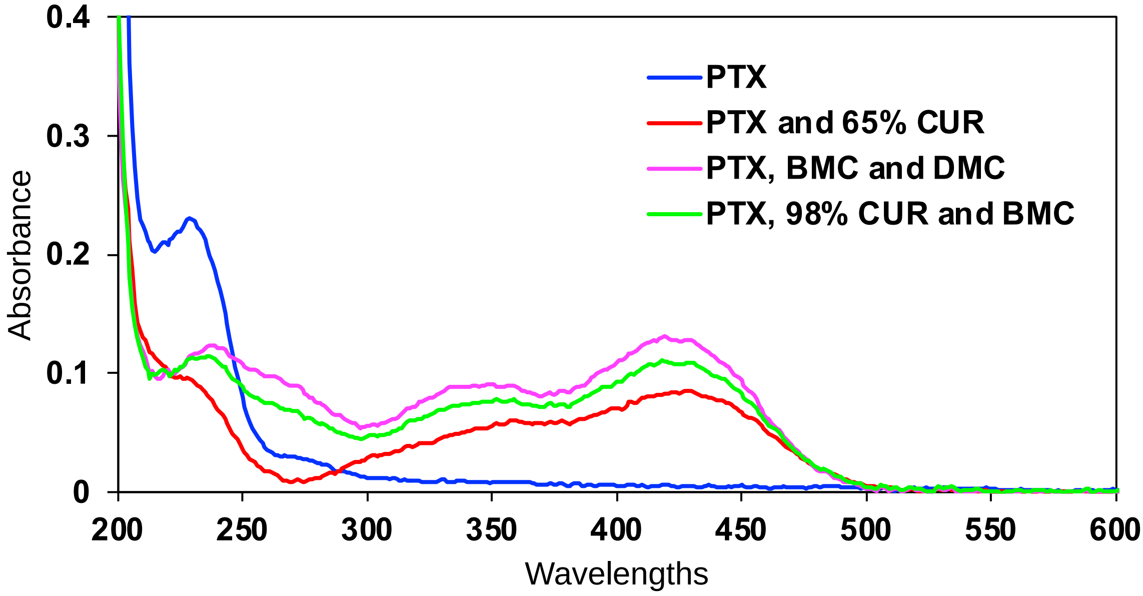

Figure S2. The UV-Vis absorption spectra of free PTX and the synthesized NPs from mixtures of PTX and 65% CUR (1), PTX, BMC and DMC (5), PTX, 98% CUR and BMC (6). PTX, CUR, BMC and DMC stand for paclitaxel, curcumin, bisdemethoxycurcumin and demethoxycurcumin, respectively.
